# *Plasmodium falciparum* Field Isolates from South America Use an Atypical Red Blood Cell Invasion Pathway Associated with Invasion Ligand Polymorphisms

**DOI:** 10.1371/journal.pone.0047913

**Published:** 2012-10-31

**Authors:** Mary Lopez-Perez, Elizabeth Villasis, Ricardo L. D. Machado, Marinete M. Póvoa, Joseph M. Vinetz, Silvia Blair, Dionicia Gamboa, Sara Lustigman

**Affiliations:** 1 Molecular Parasitology, Lindsley F. Kimball Research Institute, New York Blood Center, New York City, New York, United States of America; 2 Malaria Laboratory, Instituto de Medicina Tropical “Alexander von Humboldt” Universidad Peruana Cayetano Heredia, Lima, Peru; 3 Center for Microorganism Investigations, Department of Dermatology, Parasitic and Infectious Diseases, Medicine School in São José do Rio Preto, São Paulo State, Brazil; 4 Seção de Parasitologia, Instituto Evandro Chagas, Belém, Pará, Brazil; 5 Division of Infectious Diseases, Department of Medicine, University of California San Diego, La Jolla, California, United States of America; 6 Malaria Group, Sede de Investigación Universitaria, Universidad de Antioquia, Medellín, Colombia; 7 Departamento de Ciencias Celulares y Moleculares, Universidad Peruana Cayetano Heredia, Lima, Peru; Weill Cornell Medical College, United States of America

## Abstract

Studies of *Plasmodium falciparum* invasion pathways in field isolates have been limited. Red blood cell (RBC) invasion is a complex process involving two invasion protein families; Erythrocyte Binding-Like (EBL) and the Reticulocyte Binding-Like (PfRh) proteins, which are polymorphic and not fully characterized in field isolates. To determine the various *P. falciparum* invasion pathways used by parasite isolates from South America, we studied the invasion phenotypes in three regions: Colombia, Peru and Brazil. Additionally, polymorphisms in three members of the EBL (EBA-181, EBA-175 and EBL-1) and five members of the PfRh (PfRh1, PfRh2a, PfRh2b, PfRh4, PfRh5) families were determined. We found that most *P. falciparum* field isolates from Colombia and Peru invade RBCs through an atypical invasion pathway phenotypically characterized as resistant to all enzyme treatments (NrTrCr). Moreover, the invasion pathways and the ligand polymorphisms differed substantially among the Colombian and Brazilian isolates while the Peruvian isolates represent an amalgam of those present in the Colombian and Brazilian field isolates. The NrTrCr invasion profile was associated with the presence of the PfRh2a pepC variant, the PfRh5 variant 1 and EBA-181 RVNKN variant. The *ebl* and *Pfrh* expression levels in a field isolate displaying the NrTrCr profile also pointed to PfRh2a, PfRh5 and EBA-181 as being possibly the major players in this invasion pathway. Notably, our studies demonstrate the uniqueness of the Peruvian *P. falciparum* field isolates in terms of their invasion profiles and ligand polymorphisms, and present a unique opportunity for studying the ability of *P. falciparum* parasites to expand their invasion repertoire after being reintroduced to human populations. The present study is directly relevant to asexual blood stage vaccine design focused on invasion pathway proteins, suggesting that regional invasion variants and global geographical variation are likely to preclude a simple one size fits all type of vaccine.

## Introduction

Malaria remains an important public health problem in the developing world. In 2010, there were an estimated 216 million cases of malaria worldwide, of which 91% were due to *Plasmodium falciparum* [1]. While the vast majority of malaria cases occur in sub-Saharan Africa, the disease is a public health problem in more than 109 countries [1]. Malaria transmission in South America, typically characterized as hypoendemic and unstable, places approximately 170 million inhabitants on the continent at risk of malaria infection [1]. Sixty percent of the malaria cases occur in Brazil (Amazonian region) and the remaining 40% are distributed among 20 other Central and South America countries [2]. It has been proposed that European colonization led to the introduction of *P. falciparum* to South America. Parasite populations have been subdivided into two main genetic clusters: northern (Colombia) and southern (French Guiana, Brazil, and Bolivia). The Peruvian populations of *P. falciparum* seem to be an admixture of both [3], containing a limited number of genotypes and low recombination frequencies [4]. Notably, *P. falciparum* was reintroduced to Peru in the 1990s reaching epidemic levels after 1995 [5]; it was eradicated in Peru by the late 1980s with no new cases reported until the early 1990s [6], [7].

In humans, malaria pathogenesis is related to asexual blood parasite stages, which proliferate starting with merozoite invasion of red blood cells (RBC). Because merozoite attachment and invasion is a multi-step process mediated by a series of specific interactions between RBC receptors and parasite ligands [8], invasion-related proteins are attractive candidates for a *P. falciparum* blood stage vaccine. Two families of parasite ligand proteins –Erythrocyte-Binding Like (EBL) and the Reticulocyte-Binding homolog (RBL or PfRh) – have been shown to play essential roles in RBC invasion via multiple invasion pathways, thus providing the parasites with increased versatility to invade a broad range of RBC [8]–[10]. Members of both families bind to RBC receptors using patterns traditionally characterized by their distinctive sensitivities to trypsin and chymotrypsin (remove RBC protein receptors) and neuraminidase (remove RBC surface sialic acids) treatments. The sialic acid (SA)-dependent *P. falciparum* EBL invasion ligand family include erythrocyte-binding antigen 175 (EBA-175) [11], [12], EBA-181 (or JESEBL) [13], [14], EBA-140 (BAEBL or EBP-2) [15]–[17] and EBL-1 [18]. EBA-175 and EBA-140 bind to glycophorin A and C (GPA and GPC), respectively, in a SA- and trypsin-dependent, but chymotrypsin-resistant manner [19]–[21]. EBA-181 binds to unidentified receptor E, which is resistant to trypsin but sensitive to chymotrypsin treatment [13] and distinct from glycophorin B (GPB), the receptor of EBL-1 [18], [22]. Notably, polymorphisms identified in the binding domain of EBA-140 and EBA-181 were shown to have specific functional significance as they altered receptor specificity [14], [23], [24]. Although high levels of polymorphism were identified in the binding domain of EBA-175 [24], no association with invasion [25] or RBC binding [26] has been reported. Remarkably, EBL-1 is not expressed by all laboratory strains [27], [28] or field isolates [29], suggesting that it is not an essential invasion pathway for most parasites.

The PfRh protein family of invasion ligands is comprised of five members; PfRh1 [30], PfRh2a and PfRh2b [31]–[33], PfRh4 [34], [35] and PfRh5 [36], [37], all of which, with the exception of PfRh5, are transmembrane proteins. Only receptors for PfRh4 and PfRh5 have been identified; the complement receptor 1 (CR1) [38] and the Ok blood group antigen basigin [39], respectively. Binding to both receptors is SA-independent, and the RBC binding of PfRh5 is resistant to trypsin and chymotrypsin treatments [36], [37]. Although the identity of the other PfRh receptors are unknown, their binding profiles have been characterized: PfRh1 binds to a neuraminidase-sensitive putative receptor Y [30], [40], while the related proteins PfRh2a and PfRh2b, arising from different genes with unique 3′ domains [32], have different RBC binding profiles. PfRh2a undergoes multiple processing steps, with each product showing a unique binding profile to RBCs [41]. In comparison, PfRh2b has been shown to function in merozoite invasion via a putative receptor Z that is sensitive to chymotrypsin, but resistant to neuraminidase and trypsin treatment [33]. Polymorphisms in the PfRh proteins [42], [43] and their putative association with particular RBC invasion profiles have been reported for some members of the family [44]–[46].

Studies of RBC invasion by *P. falciparum* have been limited to field isolates from India, Africa and Mato Grosso, Brazil [25], [45], [47]–[51]. Similarly, only few studies have examined polymorphisms in EBL [25], [26], [48] and PfRh ligands [44]–[46], [48], [52] in *P. falciparum* field isolates and their association with invasion profiles. Moreover, to the best of our knowledge, no study has yet examined PfRh5 polymorphisms in field isolates. To begin to understand the geographic differentiation of *P. falciparum* invasion, phenotypically and genetically, we studied invasion phenotypes of Colombian, Peruvian and Brazilian field isolates into enzyme-treated RBCs, and compared them to those described in Africa and India. Polymorphisms in three members of the EBL (EBA-181, EBA-175 and EBL-1) and five members of the PfRh (PfRh1, PfRh2a, PfRh2b, PfRh4, PfRh5) families were determined. To date, this study is the first to simultaneously examine polymorphisms in all PfRh ligands in field isolates. These analyses confirmed that Peruvian isolates use a combination of invasion pathways likely representing an amalgam of pathways derived from Colombian and Brazilian isolates. Notably, our studies demonstrate the uniqueness of the *P. falciparum* field isolates from South America as related to their invasion profiles and ligand polymorphisms in comparison to those described in Africa and India, raising important questions about the ability of the parasites to expand their invasion repertoire after being reintroduced into local human populations.

## Results

### *P. falciparum* Field Isolates have an Atypical and Unique Invasion Pathway in Colombian and Peruvian Isolates

**Figure 1:**
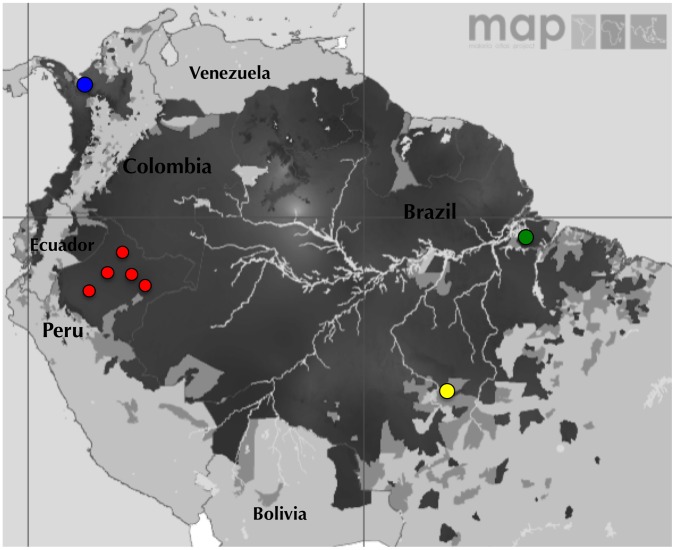
*Plasmodium falciparum* malaria endemicity in South America and geographic location of the sampling areas. The *P. falciparum* malaria endemicity for 2010 in South America is stratified according to API into stable transmission (dark grey areas; PfAPI ≥0.1 per 1000 per annum), unstable transmission (medium grey areas; PfAPI<0.1 per 1,000 per annum) and no risk or malaria free (light grey and white areas; PfAPI = 0) [81]. Circles represent populations sampled for this study: Urabá, Colombia (blue circles); Loreto, Peru (red circles); Belém and Mato Grosso, Brazil (green and yellow circles, respectively).

Invasion assays were performed with 30 field isolates from Colombia, Peru and Belém, Brazil (Figure 1). Only isolates with a single detectable clone, as determined by *msp-1* and *msp-2* genotyping [48] (data not shown), were used in the invasion assays. Controls were laboratory strains 3D7, 7G8, HB3, Dd2 and W2mef with known different invasion profiles. Primary analysis of field isolates invasion of enzyme-treated RBCs showed a broad range of sensitivity to neuraminidase (9 to 87%), trypsin (0 to 77%) and chymotrypsin (8 to 109%) with differences according to geographic origin (Table 1). Only invasion into trypsin-treated RBCs was associated with invasion into chymotrypsin-treated RBCs (*p* = 0.037; r_s_ = 0.375; Spearman’s correlation) (Figure S1), suggesting that many parasites use simultaneously invasion pathways that are dependent on receptors resistant to both enzymes. Invasion into neuraminidase-treated RBCs was independent of the other two enzyme treatments. As the parasite multiplication rate (PMR) into untreated RBCs did not differ significantly among isolates, the efficiency of invading treated RBCs was not related to parasite fitness, but rather due to specific RBCs receptors-parasite ligand interactions.

**Table 1:** Invasion profile into RBCs treated with neuraminidase, trypsin or chymotrypsin by *P. falciparum* field isolates from South America.

				% Invasion relative to untreated RBC^c^		
Invasionprofile^a^	Isolate	Origin	PMR^b^	Neuraminidase(0.1 U/ml)	Trypsin(10 mg/ml)	Chymotrypsin(2 mg/ml)
NrTrCr	WMOSQ	Colombia	5.1±0.8	67.6±9.6	74.6±16.9	59.2±8.0
	CMC	Colombia	5.3±0.6	74.3±7.4	62.0±13.6	66.1±2.8
	CEI	Colombia	5.2±2.7	57.7±5.3	61.3±20.0	74.6±7.0
	ASF	Colombia	5.0±3.1	71.8±15.7	74.3±7.9	82.1±6.3
	F04	Peru	4.8±2.3	76.9±11.7	59.4±14.6	72.9±12.0
	F13	Peru	6.0±2.4	80.3±14.9	76.9±26.4	86.0±27.4
	F14	Peru	4.0±1.9	70.7±12.1	51.9±26.2	71.3±14.2
	F15	Peru	5.9±1.4	78.4±22.7	64.9±32.9	65.7±26.0
	F23	Peru	4.4±1.4	60.3±24.4	56.1±17.1	90.2±22.8
	F26	Peru	4.0±0.8	72.9±11.7	57.4±5.8	71.0±11.9
	F32	Peru	4.1±1.3	63.4±6.5	74.2±18.8	109.4±27.8
	279	Brazil	2.0±0.1	78.4±2.2	60.3±10.0	62.9±3.3
NrTrCs	262	Brazil	2.9±0.3	57.4±1.0	76.4±5.0	17.6±1.5
NrTsCr	7G8	Brazil	7.5±4.4	71.7±11.2	31.5±10	74.3±13
	HB3	Honduras	7.5±3.4	70.3±14.7	38.2±9.7	70.3±9.0
	F06	Peru	4.3±0.9	72.0±20.0	41.3±6.3	60.2±7.8
	F12	Peru	5.0±1.1	58.6±24.8	32.1±4.1	81.3±18.9
	F19	Peru	5.2±0.9	61.4±6.8	36.9±11.4	64.5±9.4
	F25	Peru	4.8±4.2	68.7±15.5	46.3±23.9	70.1±10.6
NrTsCs	JMU	Colombia	4.5±2.6	59.4±3.8	28.5±19.2	42.6±11.9
	516	Brazil	3.5±0.0	86.7±0.0	19.5±0.0	44.2±0.0
	526	Brazil	2.6±0.2	77.4±11.2	0.0±0.0	34.0±12.9
	1038	Brazil	2.6±0.1	73.1±29.2	22.7±3.3	24.3±4.5
NsTsCs	PC15	Peru	2.7±0.3	16.3±2.9	20.1±2.9	30.6±0.0
	PC26	Peru	3.3±0.5	22.2±15.8	18.7±11.5	16.8±11.7
	PC49	Peru	3.8±0.7	8.6±8.4	30.7±1.0	40.9±4.3
NsTrCr	Dd2	Indochina	4.2±4.6	4.2±4.6	62.3±11.2	84.3±16.7
	W2mef	Indochina	6.3±0.2	2.3±0.7	78.1±0.4	73.4±7.3
	F07	Peru	4.0±2.5	28.9±21.6	57.6±4.1	85.9±20.1
	1107	Brazil	1.2±0.0	38.0±0.0	60.8±0.0	55.7±0.0
	WMONT	Colombia	3.1±0.8	48.9±11.2	52.7±34.8	77.1±20.7
NsTrCs	BEL0509	Brazil	3.5±0.0	39.3±0.0	63.1±0.0	8.3±0.0
NsTsCr	3D7	Netherlands	3.6±1.0	40.3±19.7	35.1±7.8	77.2±9.2
	F11	Peru	5.3±3.3	42.1±5.8	32.0±18.2	63.3±10.5
	1076	Brazil	3.3±0.7	46.9±13.3	33.1±3.1	77.2±3.1
	n = 6	Colombia^d^	4.7±0.8	**63.3±9.6**	**58.9±17.1**	**66.9±14.5**
	n = 16	Peru^d^	4.5±0.9	**55.1±23.7**	47.3±17.9	**67.5±23.0**
	n = 8	Belém^d^	2.7±0.8	**62.2±19.2**	42.0±26.8	40.5±23.8
	n = 14	Mato Grosso^d^	ND	27.5±21.5	**56.4±40.9**	**95.7±34.5**

a N, neuraminidase; T, trypsin; C, chymotrypsin; s, sensitive; r, resistant.

b PMR, parasite multiplication rate was determined as (parasitemia of untreated RBCs invaded/pre-invasion parasitemia).

c Percentage of invasion into enzyme-treated RBCs was calculated as (parasitemia of enzyme-treated RBC invaded/parasitemia of untreated RBCs invaded)x100. Data are means ± standard deviations. All field isolates were assessed in at least two independent experiments.

d Means ± standard deviations of all isolates per site of study. Resistant in bold: if mean of percentage of invasion is >50% than untreated RBC, the isolates were considered to be resistant to enzyme treatment.

**Figure 2:**
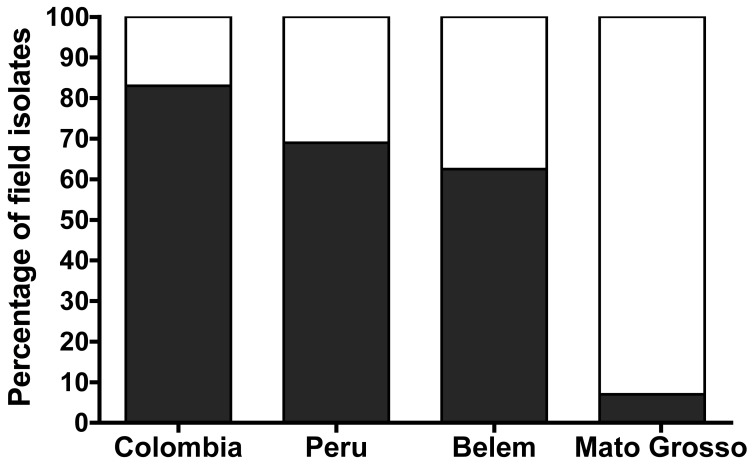
Sialic acid (SA)-dependence of invasion by *P. falciparum* field isolates from South America. Invasion via SA-dependent pathway was defined as less than 50% invasion into neuraminidase-treated RBCs. Invasion via SA-independent pathway was defined as more than 50% invasion into neuraminidase-treated RBCs. Percentage of field isolates using a SA-independent (black bars) and SA-dependent (white bars) pathway in Colombia (n = 6), Peru (n = 16) and Belém, Brazil (n = 8) or Mato Grosso, Brazil (n = 14) is compared.

*P. falciparum* invades RBCs through two major types of pathways: SA-dependent and SA-independent. Invasion via SA-independent receptors, defined as >50% invasion into neuraminidase-treated RBCs, was significantly more common in field isolates from Colombia (83%), Peru (69%) or Belém, Brazil (63%) than in Mato Grosso, Brazil (7%; *p* = 0.02, Fisher’s exact test, Figure 2) [48].

**Figure 3:**
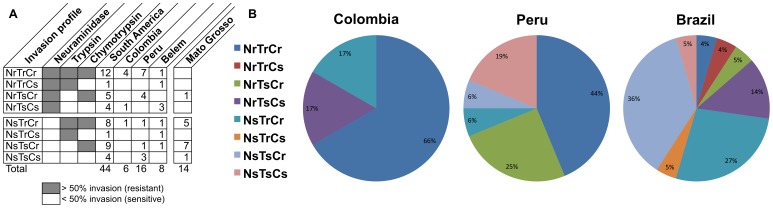
Distribution of the eight profiles of invasion in field isolates from Peru, Colombia and Brazil. (**A**) The number of isolates using a specific invasion profile based on their combined sensitivity to neuraminidase (N), trypsin (T) and chymotrypsin (C). Sensitive (s) is defined as less than 50% of invasion into enzymes-treated RBC (white box) and resistant (r) is defined as higher than 50% of invasion (gray box). (**B**) Percentage of invasion profiles in each area of study is presented using pie charts. Brazilian isolates include those from Belém and Mato Grosso.

The invasion profile of *P. falciparum* is often also described as a combination of sensitivity to three enzymes (one glycohydrolase, two proteases): neuraminidase (N), trypsin (T) and chymotrypsin (C) [25], [48], [50], [51], which can result in eight possible invasion profiles. Indeed, all eight invasion profiles were observed in field isolates from South America, with different distribution according to geographic origin (Figures 3A and 3B, Table 1). Colombian and Brazilian isolates are distinctively different, whereas Peruvian isolates seem to represent an amalgam of Colombian and Brazilian invasion profiles. The invasion profiles found in South America, including Mato Grosso, are distributed into four major groups: i) NrTrCr (12/44); ii) NsTsCr (9/44); iii) NsTrCr (8/44); and iv) NrTsCr (5/44). The invasion profile resistant to all treatment (NrTrCr) was significantly more prevalent in Colombia (4/6) and Peru (7/16) than in Brazil (1/22) (*p* = 0.002, Fisher’s exact test). The same profile was reported previously in two culture-adapted strains from Papua New Guinea (1935) and Africa (JO) [53]. The most common profile found in Mato Grosso, Brazil was NsTsCr (7/14), a pathway used by the 3D7 laboratory strain. This pathway was found in a minority of parasites from Peru (1/16) and Belém (1/8). The second most frequent invasion profile in Mato Grosso, Brazil was NsTrCr (5/14) [48], similar to the Dd2 laboratory strain; this profile also was observed in a minority of isolates from Colombia (1/6), Peru (1/16) and Belém (1/8). The invasion profile NrTsCr, known to be used by the Brazilian 7G8 and Honduran HB3 laboratory strains, was reported previously in only one field isolate from Mato Grosso and it was the second most common pathway used by Peruvian isolates (4/16). This invasion pathway was not found in Colombian isolates or in isolates from Belém. Four other but less frequent invasion profiles were also found: i) NrTsCs in field isolates from Colombia (1/6) and Belém (3/8); ii) NrTrCs (1/8) and NsTrCs (1/8), which were both exclusively found in Belém; and iii) NsTsCs, which was reported in only one field isolate from Mato Grosso [48], and in this study was also found in three Peruvian isolates (PC15, PC26 and PC49). These isolates were collected in a village of Padre Cocha near Iquitos where *P. falciparum* had not been present for many years until a small outbreak of malaria occurred in the 1990s after a person returned from Brazil to his home in Padre Cocha; thus these isolates are assumed to come originally from Brazil (Alan J. Magill, personal communication).

### Polymorphisms in EBL Ligands are Differentially Distributed in South America

Specific polymorphisms in the binding domain of EBA-140 [23] and EBA-181 [14], [23], [24] have been shown to have functional significance because they can alter receptor specificity. EBL-1 is known to have three major polymorphisms, two of which make it non-functional [28], [29]. In this study, sequence polymorphisms in EBA-181, EBA-175 and EBL-1 were analyzed in field isolates from Colombia, Peru and Belém, Brazil. Additionally, the polymorphisms in EBA-175 and EBL-1 were analyzed in the previously characterized Mato Grosso isolates [48].

**Figure 4:**
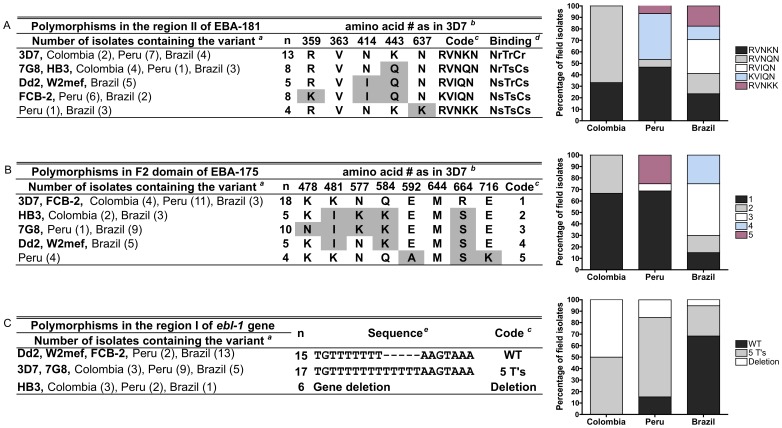
Presence and prevalence of EBL polymorphisms in field isolates from Peru, Colombia and Brazil. (**A**) Variants of EBA-181 protein were defined according the amino acid substitutions at five positions. The percentage of field isolates from Colombia, Peru and Brazil (combination of samples from Belém and Mato Grosso) with each of the variants is shown. (**B**) Amino acid substitutions at eight positions were used to categorize the EBA-175 variants. (**C**) Variants of *ebl-1* gene were defined according to the absence (WT, wild type) or presence of 5 thymidines insertion at position 572 (5 T’s), and the absence of the gene after nested PCR amplification (deletion).*^a^* Number of parasites containing the variant in each area of study is shown in parenthesis. *^b^*Amino position according to the reference sequence is shown. EBA-181 (GenBank accession no. AY496955; 3D7), EBA-175 (GenBank accession no. FJ655429; 3D7) and EBL-1 (GenBank accession no. AF131999; Dd2). Shaded residues differ from the strain reference sequence.*^c^*Code used in bar graphs and text. *^d^*The RBC binding profile reported per each EBA-181 variant is shown [14]. Neuraminidase (N), trypsin (T), chymotrypsin (C), sensitive (s) and resistant (r).*^e^* Sequence alignment of nucleotides 564–584 of the *ebl-1* gene is shown.

The sequence encoding the region II of EBA-181 (aa 108 to 774) was determined in the South American field isolates and compared to those present in six laboratory strains (3D7, 7G8, HB3, Dd2, W2mef and FCB-2) where eight variants were reported. These variants are based on amino acid substitutions in five positions 359, 363, 414, 443 and 637 according to 3D7 sequence [14]. Five of the eight EBA-181 variants previously reported in laboratory strains [14] were found in the isolates from Colombia, Peru and Belém, Brazil (Figure 4A). These variants are the same as those previously reported in field isolates from Mato Grosso [48] but their distribution, including Mato Grosso, was distinct based on parasite origin (Figure 4A). The most common RVNKN variant (3D7-like) was found in all sites (13/38); while the RVIQN variant (Dd2-like) was found exclusively in Brazil (one in Belém and 4 in Mato Grosso). The 7G8 and HB3-like RVNQN variant was only common in Colombia (4/6), but rare in Peru (1/13) and Brazil (3/17). In contrast, the KVIQN (FCB-2-like) and RVNKK (PC49-like) variants were found only in Peru and Brazil but not in Colombia.

Region II of EBA-175 (F1 and F2 domains) is more polymorphic than the other members of EBL family and appears to be under diversifying selection [24]. We analyzed the sequences in the F2 domain of EBA-175 (aa 366 to 746), and defined the variants according to amino acid at positions 478, 481, 577, 584, 592, 644, 664 and 716. Five variants in the EBA-175 sequence (Figure 4B) were found, four of which were 3D7, HB3, 7G8 and Dd2/W2mef-like (coded 1 to 4, respectively) and a new variant (variant 5) was found in Peruvian field isolates (4/16). The 3D7-like variant 1 was the most frequent (18/42) in South America and the most prevalent in Peru (11/16). The HB3-like variant 2 was found only in Colombia (2/6) and Brazil (3/20), while the 7G8-like variant 3 was the most prevalent in Brazil (9/20). Variant 4, Dd2-like, was found exclusively in Brazil, in one isolate from Belém and four from Mato Grosso.

EBL-1 is the ligand for GPB [18], [22]. Interestingly, the *ebl-1* gene in many laboratory strains and field isolates has a five-thymidine insertion at nucleotide position 572 (region I) that changes the reading frame of the gene, resulting in premature translational termination [28], [29]. Consistent with previous reports [27], [28], field isolates from Colombia, Peru and Brazil (Belém and Mato Grosso) had three known variants: i) normal *ebl-1* gene sequence similar to Dd2 strain, coded as wild type (WT); ii) *ebl-1* gene containing a 5 thymidine insertion (coded as 5 T’s) similar to 3D7 strain; and iii) complete *ebl-1* gene deletion (coded deletion) similar to HB3 strain (Figure 4C). The majority of field isolates from Colombia (3/6) and Peru (9/13) had a 5 T’s insertion, suggesting that no functional EBL-1 protein is produced. Similar results were found in Kenya, where 34/47 isolates had a 5 T’s insertion [29]. In contrast, the majority of Brazilian isolates (13/19) and only two Peruvian isolates (the PC isolates originated from Brazil) had a normal *ebl-1* gene sequence (Figure 4C). In few isolates (6/39; 3 Colombian, 2 Peruvian and 1 Brazilian isolates), no PCR products were obtained, signifying a complete gene deletion similar to the HB3 strain. These results suggest that the EBL-1 ligand does not play an indispensable role in invasion by Colombian and Peruvian isolates, but is potentially more utilized by Brazilian isolates. These finding are consistent with our previous studies showing that polymorphisms in GPB, the EBL-1 receptor, are associated with *P. falciparum* susceptibility in the Brazilian Amazon [54].

### Polymorphism in PfRh Ligands: New Variants and Differential Distribution in South American Isolates

Polymorphisms in PfRh1, PfRh2a, PfRh2b and PfRh4 have been reported previously in field isolates [44]–[46], [52]. However, to the best of our knowledge, no study has yet examined PfRh5 polymorphisms. In this study, polymorphisms in all PfRh ligands corresponding to regions already described in laboratory strains are analyzed for the first time.

**Figure 5:**
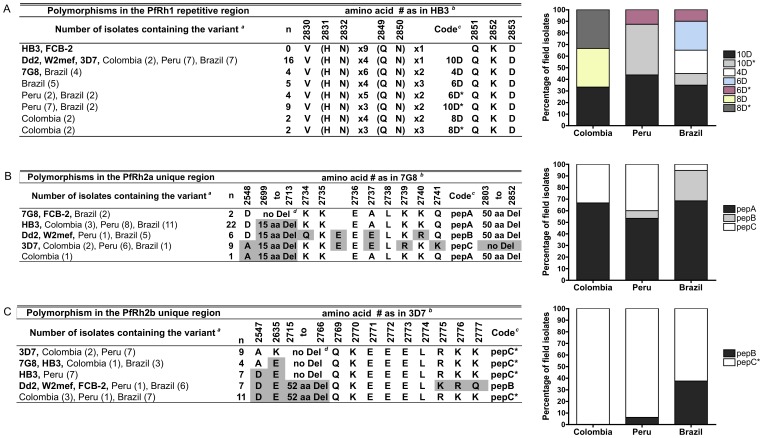
Presence and prevalence of PfRh1, PfRh2a and PfRh2b polymorphisms in field isolates from Peru, Colombia and Brazil. (A) Variants of PfRh1 were defined by the number of HN and QN repeats and amino acids deletion. The percentage of field isolates from Colombia, Peru and Brazil (combination of samples from Belém and Mato Grosso) with each of the variants is shown. (B) Haplotypes in PfRh2a were defined according to amino acid deletion (15 aa and 50 aa deletion) and the peptide sequence at position 2734–2741. (C) Variants of PfRh2b were defined according to amino acid deletion (52 aa) and the peptide sequence at position 2769–2777. *^a^*Number of parasites containing the variant in each area of study is shown in parenthesis. *^b^*Amino position according to the reference sequence is shown. PfRh1 (GenBank accession no AF411930; HB3), PfRh2a (GenBank accession no. AY138497; 7G8) and PfRh2b (GenBank accession no. AY138500; 3D7). Shaded residues differ from the strain reference sequence.*^c^*Code used in graphs and text. *^d^*Del: deletion.

Significant polymorphisms in the repetitive region of the PfRh1 (aa 2712–2870) have been reported [30], [42]. This region contains a series of repeated residues, 9×(HN) repeat near the C-terminal followed by 1×(QN) repeats based on the sequence of the HB3 reference strain. The number of these two dipeptide repeats differed among the isolates, resulting in four to ten amino acids deletion. We found seven PfRh1 variants in the field isolates from Colombia, Peru and Brazil, coded according to the number of amino acid deleted (Figures 5A). Two novel variants with eight amino acid deletions (coded 8D and 8D*) were found in two Colombian isolates each. The majority of the isolates (16/42) had a variant reported in Dd2 and 3D7 (coded 10D, 10 aa deletion). Parasites with 10 aa deletion in PfRh1 were the most common in Peru (7/16, variant 10D or 10D*). The variants 6D (5/20) and 4D (4/20) were found exclusively in Brazil, while variant 6D* was found in Peru (2/16) and Brazil (2/20). The HB3-like variant (no aa deletion) was not found in any of the field isolates from South America, except in the Colombian FCB-2 laboratory strain.

The polymorphisms in the PfRh2a and PfRh2b related proteins [32] were performed for the repeat and 3′ unique regions of PfRh2a (aa 2379 to 3117) and PfRh2b (aa 2379 to 3141). The haplotypes in PfRh2a were defined according to an amino acid substitution at position 2548 (D2548A), a 15 amino acid deletion starting at position 2699, a 50 amino acid deletion starting at position 2803 and the sequence at aa 2734–2741 (Figure 5B), which is within the PfRh2a peptide sequence (peptide 26835 in 3D7, LEREKQEQLQKEEELKRQEQY) shown to bind to RBCs [55]. Three peptide variants were identified, which were named pepA (KK-EALKKQ), pepB (QKEEELKRQ) or pepC (KKEEELRKK). Five haplotypes of PfRh2a were found; four of which are similar to laboratory strains (Figure 5B). The most common haplotype was the HB3-like (22/40), followed by the 3D7-like (9/40). Two isolates from Brazil, and the Colombian FCB-2 laboratory strain, were similar to the Brazilian 7G8 laboratory strain haplotype. Notably, a new haplotype was found in one of the Colombian field isolates. The majority of isolates (31/40) from South America contained the 50 amino acid deletion at position 2803 in contrast to 1/17 of Senegalese isolates [45]. The peptide variants defined as pepA, pepB and pepC had different distribution in isolates from Colombia, Peru and Brazil (Figure 5B). The majority of parasites (25/40) from the three countries contained the polymorphic region identified as pepA, 4/6 in Colombia, 8/16 in Peru and 13/19 in Brazil. The pepC variant was found in 9/40; 6 Peruvian, 2 Colombian and one isolate from Belém, Brazil. In contrast, pepB was found in five isolates from Mato Grosso, Brazil and only in one Peruvian isolate. In comparison, among Senegalese field isolates only pepA and pepC variants were reported [45].

Haplotypes in PfRh2b were defined according to amino acid substitutions at positions 2547 (A2547D) and 2635 (K2635E), a 52 amino acid deletion starting at position 2715 and the nine amino acid peptide sequence at position 2769–2777. Two peptide variants were found; pepB (QKEEELKRQ), similar to Dd2, and pepC* (QKEEELRKK), similar to the variant found in 3D7 and related to pepC present in PfRh2a. Analysis of the PfRh2b sequences in the South American isolates revealed five haplotypes (Figure 5C). Four are similar to those already found in laboratory strains and one variant is similar to the novel variant found in the GVM field isolate from Mato Grosso, Brazil [44]. The most common haplotype was the GVM-like (11/38), followed by the 3D7-like (9/38) and the HB3-like (7/38), none of which were described previously in Mato Grosso isolates [44]. Only 13% of the isolates from Peru contained a 52 amino acid sequence deletion found in Dd2; similar to what was reported in the isolates from Senegal [45]. In contrast, this deletion was found in 50% of the Colombian isolates and 56% of the Brazilian. The pepC* variant was the most frequent (31/38) in all three South American countries (Figure 5F). Parasites with PfRh2b pepC* variant can be divided into two groups; with or without the 52 aa deletion 5′ to the peptide. Notably, parasites containing the pepC* variant without the deletion were more common in Peru (14/16), while pepC* plus the deletion (GVM-like) variant was more common in Mato Grosso (7/14). The pepB variant was present in six Brazilian (two from Belém and four from Mato Grosso) and only one Peruvian isolate.

A large deletion of 585-bp in the PfRh2b unique region downstream of the repetitive region starting at amino acid position 2941 was reported in the T996 laboratory strain from Thailand [56] and in 64–68% of field isolates from Senegal [45], [46]. This deletion was also reported to be highly frequent in other four sites; Senegal (63–72%), Tanzania (87%), Malaysia (84%) and Malawi (58%). However, its presence was less frequent in Thailand (10%) and Brazil (8%) [52]. In our study, this deletion was observed in only 8/40 field isolates (20%); one from Colombia (17%) and seven from Peru (44%). In contrast, all isolates from Brazil (Mato Grosso and Belém), and the six laboratory strains from diverse geographical locations, which we analyzed, contained the full-length sequence of PfRh2b.

**Figure 6:**
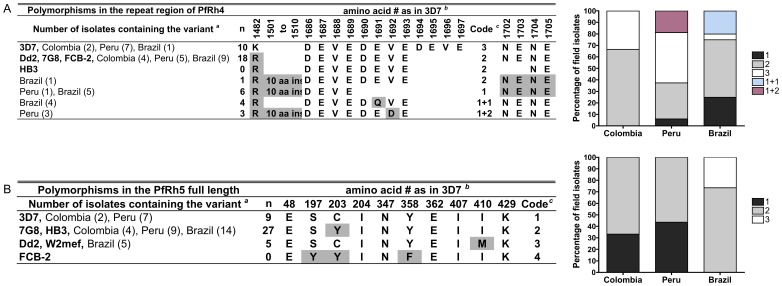
Presence and prevalence of PfRh4 and PfRh5 polymorphisms in field isolates from Peru, Colombia and Brazil. (**A)** Variants of PfRh4 were defined by the number of DEVE repeat. The percentage of field isolates from Colombia, Peru and Brazil (combination of samples from Belém and Mato Grosso) with each of the variants is shown. (**B**) Haplotypes in PfRh5 were defined as combination of amino acids in 10 positions reported previously as polymorphic [43]. *^a^*Number of parasites containing the variant in each area of study is shown in parenthesis. *^b^*Amino position according to the reference sequence is shown. PfRh4 (GenBank accession no. AF432854; 3D7); PfRh5 (GenBank accession no. PFD1145c). Shaded residues differ from the strain reference sequence.*^c^*Code used in graphs and text. *^d^*Ins: insertion.

ThePfRh4 protein (1716 amino acids in the 3D7), contain two repeat regions; at amino acids 1494**–**1543 [H(T/A)NE(K/N)NI(N/Y)(N/Y)E×5] within the extracellular domain and at amino acids 1686–1712 [(D/N/V)E×13] within the cytoplasmic domain [34]. Analysis of these sequences in our field isolates show five variants according to the number of copies and modifications of the tetrapeptide repeat DEVE (aa 1686–1697) and an amino acid substitution at position 1482 (K/R). Two variants were 3D7-like or 7G8/Dd2/W2mef-like and none were HB3-like (Figure 6A). Two other variants (coded 1 and coded 1+1) were similar to those already reported in the field isolates from Mato Grosso [44]. The fifth variant is new and found in only the three Peruvian Padre Cocha isolates thought to be of Brazilian origin because of direct evidence of importation. This variant contains one DEVE repeat followed by a modified tetrapeptide DEDE followed by NENE (code 1+2). Notably, the South American variants had a novel polymorphic region in the PfRh4 sequence; a 10 aa insertion (HTNENNINNE) starting at position 1501 that was present in ten isolates (four from Peru, one from Belém and five from Mato Grosso). Parasites with Dd2/7G8/FCB-2-like variant containing two DEVE repeats (code 2) were the most frequent (18/42) in South America (Figure 6A). This variant was also the most prevalent in Colombia (4/6) but not in Peru (5/16). The 3D7-like variant (code 3) was predominant in Peru (7/16), and was less represented in Colombia (2/6) or Brazil (1/20). Parasites containing one DEVE repeat followed by a modified tetrapeptide DQVE/DEDE (code 1+1 or 1+2, respectively) were found in low frequency in Brazilian isolates (4/20) and in all the Peruvian PC isolates (3/16), which originated from Brazil.

Sequencing of PfRh5 full length in 18 laboratory strains from diverse geographic origin has shown limited polymorphism in this protein; amino acid substitutions in 10 of 526 amino acids have been found, all of which represent eight distinct variants [43]. Whether these variants are random or due to immune selection is not known. Nonetheless and importantly, a change in one amino acid at position 204 (I204K) altered RBC binding hence receptor recognition [43]. Four of the eight PfRh5 variants reported [43] were found in field isolates from South America, one of which was only found in the FCB-2 Colombian laboratory strain (Figure 6B). Interestingly, this haplotype is similar to the Geneva laboratory strain from Senegal [43], which grows in *Saimiri sciureus* monkeys [57]. The most common haplotype was the 7G8/HB3-like (27/41) found in all study sites (Figure 6B). In contrast, the 3D7-like haplotype was found only in isolates from Colombia and Peru (9/41). The Dd2-like variant was the least frequent (5/41) and was only found in Brazilian field isolates, one from Belém and four from the newly analyzed Mato Grosso isolates. In summary, limited polymorphism in the PfRh5 ligand was found and only two amino acids (aa position 203 and 410) of the ten reported were found in South American field isolates. Notably, one of these polymorphisms, a Cys to Tyr residue change at aa position 203 (C203Y) was found in 27/41 field isolates, which may have significant structural and functional implications as previously suggested [43].

### Association of Polymorphisms in EBL Proteins with Invasion Pathways

Polymorphisms in region II of EBA-181 alter the binding profile of the variants to enzyme-treated RBCs [14] or affect their level of binding to RBCs [26]. Polymorphism in EBA-175 has not been associated with differing RBC binding patterns [26] or invasion profiles by the Gambian field isolates [25]. In our study we found that the RBC invasion by parasites expressing the EBA-181 variant RVIQN were highly sensitive to neuraminidase and resistant to trypsin-treatment with RBC invasion rates of 3.6% and 76%, respectively. In contrast, parasites containing other EBA-181 variants had more frequent invasion of neuraminidase-treated RBCs (median 43–71%) and less frequent invasion of trypsin-treated RBCs (median 38–51%). Using Principal Component Analysis (PCA), 6 of 7 isolates containing the RVIQN variant, all from Mato Grosso, cluster at the bottom right quadrant of the PCA projection (Figure S2B) suggesting a significant association between the RVIQN variant and NsTrCr invasion profile (*p*<0.0001, Fisher’s exact test). Notably, this variant was also associated with an NsTr RBC binding profile [14] and the lowest binding to untreated RBCs (∼40% of the control) in comparison to binding by other variants of the protein [26]. Parasites containing the RVNKN variant were clustered at the top left quadrant (9/14). Analysis of association between the presence of this variant with the NrTrCr invasion profile revealed borderline significance *(p* = 0.056, Fisher’s exact test) suggesting that it will be necessary to analyze more isolates to confirm a significant association. Notably, the RVNKN variant has been reported to be associated with the NrTrCr RBCs binding profile [14].

The NsTsCr profile of the EBA-175/GPA invasion pathway was found only in one isolate from Peru, one from Belém and seven from Mato Grosso reported previously [48]. Only two of the Mato Grosso seven parasite isolates invaded cells lacking GPA [En (a**-**)], while the others invaded with efficiencies ranging 50–92% [48], suggesting that these parasites do not necessarily use the EBA-175/GPA pathway for invasion. Interestingly, analyze of the anti-EBA-175 and EBA-140 antibody responses in individuals from Mato Grosso, have shown that they have a much lower IgG responses as compared to those in Cameroon, Africa. We, therefore, proposed that in this region the parasites depend more on GPB and/or other invasion pathways that use NsTs/rCs/r RBC [58]. The newly studied parasites with Dd2-like EBA-175 variant 4 were found to be associated with an invasion that is highly sensitive to neuraminidase and resistant to trypsin and chymotrypsin, NsTrCr (*p*<0.0001, Fisher’s exact test). However, the meaning of this association is unknown (Figure S2C) as the identity of the particular receptor(s) of Dd2 invasion pattern is not yet known. Future growth inhibition assay studies using anti-EBA-175 antibodies against South American isolates and isolates from other regions of the world, would clarify to what extent the EBA-175/GPA pathway is utilized by the South American NsTs/rCs/r parasites.

The GPB has been identified as the RBC receptor of EBL-1 with a binding profile that is sensitive to neuraminidase and chymotrypsin, but resistant to trypsin (NsTrCs) [18], [22]. However, the importance of EBL-1 for RBC invasion is unclear [28], [29]. Our previous studies in Brazil have suggested an important role for the GPB receptor in RBC invasion by *P. falciparum* Brazilian isolates based on a high prevalence of GPB S+ variant in *P. falciparum* infected individuals and the GPB s- variant in uninfected individuals [54]. Here we found that the wild type (WT) fully expressed EBL-1 ligand was found mainly in the isolates from Brazil including 2/3 of the Padre Cocha isolates from Peru that originated from Brazil. Notably, the *ebl-1* gene sequence containing the 5 T’s insertion was more frequent in parasites with the NrTrCr invasion profile (*p* = 0.020, Fisher’s exact test), frequent in Colombian and Peru (Figure S2D). Thus, it is probable that the parasites from Brazil use the EBL-1/GPB pathway while the parasites from Colombia and Peru are not dependent on this pathway. To address the possible involvement of GPB-dependent invasion in field isolates from South America, trypsin-treated GPB deficient (S-s-U-) RBCs were used in invasion assays with some field isolates. Field isolates from Colombia and Peru (except PC isolates) invaded trypsin-treated S-s-U- RBCs at invasion rates of 40% (SD ±8%) and 34% (SD ±12%) relative to untreated S-s-U- RBCs, respectively. In contrast, the Peruvian Padre Cocha isolates (originally from Brazil) had low invasion rates (19±13%) while field isolates from Belém, Brazil were unable to invade trypsinized S-s-U- RBCs (1±2%). Hence, the absence of GPB reduced the invasion of trypsin-treated RBC by >80% in both Padre Cocha and Belém isolates, suggesting that GPB plays a role as an RBC receptor by these isolates but not in parasites from Colombia and Peru. Unfortunately, we could not test the use of GPB as a receptor for the isolates from Mato Grosso, Brazil, as the assays were performed in Brazil at a time it was impossible to find S-s-U- RBCs locally [48].

### Association of Polymorphisms in PfRh Proteins with Invasion Pathways

There are few published data on association of PfRh protein polymorphism with parasite invasion profiles using field isolates [44]–[46], [48]. Our present analysis found associations between specific variants of the PfRh proteins and invasion profiles of South American field isolates.

As described above, the PfRh1 polymorphisms correspond to the number of copies of HN and QN, which could result in 4 to 10 amino acid deletions (Figure 5A). The 10 aa deletion (10D and 10D* codes) was the most common polymorphism among the isolates studied here, and was found to be significantly associated with an invasion that uses trypsin and chymotrypsin resistant receptor (*p* = 0.009, Fisher’s exact test), but not with an invasion profile indicating sensitivity to neuraminidase treatment (Figure S3B) as was reported in 14 samples from Mato Grosso [44]. The differences observed with our first study are possibly due to more field isolates from different origins analyzed this time. Notably, in Senegal no significant association was found between this particular variant and invasion into enzyme-treated RBC [45].

One of the polymorphisms in PfRh2a and PfRh2b was defined based on a peptide sequence at position 2734–2741 shown to bind to RBCs [55]. The variants identified within this peptide region in PfRh2a were named pepA (KK-EALKKQ), pepB (QKEEELKRQ) and pepC (KKEEELRKK) in PfRh2a (Figure 5B). In PfRh2b, two variants were found; pepB (QKEEELKRQ) and pepC* (QKEEELRKK) (Figure 5C). The pepB variant of both PfRh2a and PfRh2b has been associated with neuraminidase sensitive and trypsin resistant invasion (NsTr) in the previously characterized Brazilian isolates from Mato Grosso [44]. The invasion of parasites from South America containing the PfRh2a pepC variant was associated with resistance to neuraminidase and trypsin-treatment with a median of 71% and 59% invasion rates, respectively. In contrast, the invasion of parasites expressing the pepB variant was highly sensitive to neuraminidase treatment (4%) and predominantly resistant to trypsin treatment (62%). Using PCA, 7/8 parasites containing the pepB clustered at the bottom right quadrant as reported for Mato Grosso isolates while 8/10 of parasites expressing pepC variant were clustered in the top left quadrant (Figure S3C). Significant associations between NsTrCr invasion profile and pepB *(p* = 0.0003, Fisher’s exact test) and NrTrCr profile with pepC (*p* = 0.007, Fisher test) were observed. Interestingly, parasites expressing pepB variant were more common in Mato Grosso isolates, in which the NsTr invasion profile was the second most frequent (5/14), while the pepC variant was almost exclusively expressed in Colombian and Peruvian isolated that use mostly the NrTrCr invasion pathway.

Only two peptide variants were found within the PfRh2b protein; pepB and pepC* (Figure 5C). Invasion of parasites expressing the pepB variant was sensitive to neuraminidase treatment (4% invasion) and resistant to trypsin treatment (63%). This variant was also found to be strongly associated with the NsTrCr invasion pathway (*p* = 0.0009, Fisher’s exact test) (Figure S3D). The analysis also revealed an association between the expression of pepC* variant and a trypsin sensitive invasion pathway that was independent of the sensitivity to neuraminidase or chymotrypsin treatment [Nr/sTsCr/s*; p* = 0.021, Fisher’s exact test)], similar to what was reported for isolates from Mato Grosso [44]. When the parasites expressing pepC* were divided into two groups based on PfRh2b variants containing the haplotype with or without the 52 aa deletion (Figure 5C), only an association between pepC* plus the deletion (GVM-like) and NsTsCr invasion profile was obtained (*p* = 0.0007, Fisher’s exact test). The other large deletion in PfRh2b (194 amino acids in the C-terminal end) was reported to be associated with enhanced invasion into trypsin-treated RBC by Senegalese field isolates [46]. However, no association was found between this particular deletion and invasion into enzyme-treated RBCs by the South American field isolates.

The most important PfRh4 polymorphisms are the number of copies or modification of the DEVE repeat and the 10 amino acids insertion unique to South American isolates (Figure 6A). When these specific polymorphisms were used to test association with the corresponding invasion profiles (Figure S3E), parasites with modified DEVE variants (coded 1+1 or 1+2) were significantly associated with an invasion phenotype of neuraminidase, trypsin and chymotrypsin sensitivity [NsTsCs; (*p* = 0.0002, Fisher’s exact test)]. Association between NsTs invasion regardless of its sensitivity to chymotrypsin (NsTsCr/s) was also observed in parasites expressing PfRh4 with a 10 aa insertion (*p* = 0.0039, Fisher’s exact test). Similar analysis in Mato Grosso isolates did not find such associations, probably because too few parasites had these specific polymorphisms [44].

Two specific PfRh5 haplotypes, defined as the particular combination of amino acids in the 10 previously reported polymorphic positions found in South American isolates (Figure 6B), variant 3 and variant 1, were associated with invasion profiles (Figure S3F). The median percentage of invasion by parasites expressing with the Dd2-like variant 3 was significantly lower in neuraminidase-treated RBCs (3.6%) and significantly higher in trypsin-treated RBCs (76%). Using PCA, variant 3 was significantly associated with the NsTrCr invasion profile *(p*<0.0001, Fisher’s exact test) whereas the 3D7-like variant 1 was associated with neuraminidase and chymotrypsin resistant invasion profile regardless of its sensitivity to trypsin treatment [NrTs/rCr; (*p* = 0.009, Fisher’s exact test)].

Analysis based on only a single polymorphism in amino acid 203 (C203Y variant), have indicated that parasites expressing all six cysteine residues in PfRh5 (Cys203 variant), such as the 3D7 and Dd2 laboratory strains, had a higher median rate of invasion into trypsin-treated RBCs (62%) than those containing only five cysteine residues (Tyr 203 variant) such as observed in the 7G8 laboratory strain (41%). Parasites containing isoleucine at amino acid position 410 (Ile 410 variant) had a higher median rate of invasion into neuraminidase-treated RBCs (59%) and intermediate rate of invasion into trypsin-treated RBCs (42%). Moreover, it appears that parasites expressing the combined PfRh5 haplotype of Cys 203 and Ile 410 are more associated with the NrTrCr/s invasion profile, when compared to those who express Tyr 203 and Met 410 variants.

### Coordinate Expression of the *ebl* and *Pfrh* Transcripts in Isolates that Use the NrTrCr Invasion Pathway

**Figure 7:**
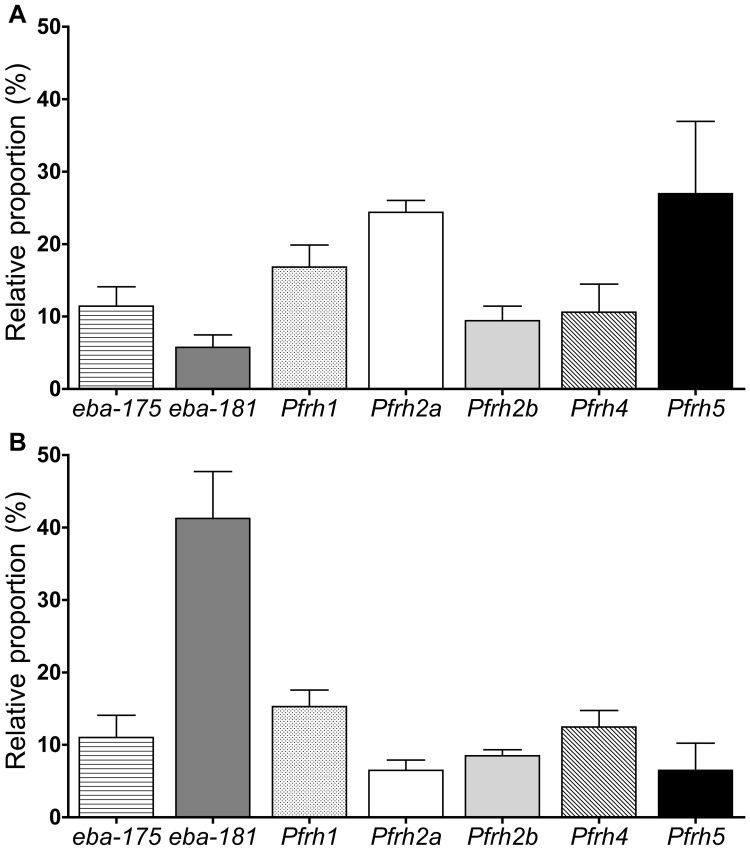
*Pfrh2a*, *Pfrh5* and *eba-181* are the most abundantly expressed ligand transcripts infield isolates utilizing the NrTrCr invasion profile. Each bar shows the relative proportion value for each transcript as a percentage of all seven transcripts including *eba-175*, *eba-181*, *Pfrh1*, *Pfrh2a*, *Pfrh2b*, *Pfrh4* and *Pfrh5*. Values are given as means ± standard errors of the means. (A) Comparison of the transcriptional levels of the ligands between parasites utilizing NrTrCr and NsTrCr invasion profiles. (B) Comparison of the transcriptional levels of the ligands between parasites utilizing NrTrCr and NrTsCr invasion profiles.

The most common invasion profile observed in Colombian and Peruvian isolates was NrTrCr, which is a very rare profile in field isolates, described so far. To obtain an indirect indication of parasite ligands possibly involved in the NrTrCr invasion pathway, the expression of all *ebl* and *Pfrh* transcripts was analyzed in four NrTrCr isolates. For comparison we used two field isolates exhibiting common invasion profiles and in which only one change in the sensitivity to enzyme-treatment was present: F07, sensitive only to neuraminidase (NsTrCr); and F25, sensitive only to trypsin (NrTsCr). The low frequency of isolates that are sensitive to chymotrypsin treatment in South America precluded a comparison to the NrTrCs profile. Analysis of the fold change expression levels in the NrTrCr isolates in comparison with the field isolates F07 and F25 showed ≥5-fold increased expression in all genes tested. *Pfrh2a* and *Pfrh5* were the most abundant transcripts (relative proportion of 24% and 22%, respectively) compared to F07 (Figure 7A) and *eba-181* was the most abundant transcript compared to the NrTsCr isolate F25 (relative proportion of 41%; Figure 7B). All other transcripts had similar mean proportions (5 to 17%) of expression levels (Figure 7A and 7B). Our small sample size does not allow one to draw definitive conclusions, but nonetheless, there were significant associations between NrTrCr invasion profiles and ligand polymorphisms for the same ligands that exhibited higher gene expression levels (EBA-181, PfRh2a, and PfRh5). Notably, an NrTrCr binding profile has been reported for PfRh5 [31], [32]. The NrTrCr/s binding profile has been described for the 140 kDa-processed fragment of PfRh2a, which was shown to play an important role in the invasion process [41]. The NrTrCr binding profile has been associated with the EBA-181 RVNKN variant [14]. Taken together, these results suggest that these three ligands contribute to the NrTrCr invasion phenotype.

## Discussion

Here we report the unique finding that most *P. falciparum* field isolates from Colombia and Peru invade RBCs through an atypical invasion pathway phenotypically characterized as resistant to neuraminidase, trypsin and chymotrypsin treatment (NrTrCr). Additionally, significant level of polymorphisms in the PfRh and EBL proteins was found in these isolates including some novel polymorphisms. Moreover, the parasite invasion and ligand polymorphisms differed substantially among the Colombian and Brazilian isolates while the Peruvian isolates represented an amalgam of the most common invasion profiles and polymorphisms found in the parasites from both countries. These findings are consistent with recent genotyping observations indicating that Peruvian parasites seem to be an admixture of Colombian and Brazilian parasite populations [3]. Based on our observations we propose that *P. falciparum* strains from South America, introduced during European colonization [3], present a unique opportunity to study the geographic differentiation of these parasites with regard to their invasion phenotypes and associated ligand polymorphisms. Moreover, the newly reintroduced *P. falciparum* strains in the Peruvian Amazon have an expanded invasion repertoire, which utilizes also novel ligand-receptor interactions. Such geographic differentiation, we hypothesize, might be driven by either founder effect or by parasite genetic factors and/or human humoral immunity.

Studies of invasion pathways used by field isolates have been limited to few regions and the majority of them were done on parasites obtained in African countries [25], [45], [47]–[51]. Similar to the results of a previous study in Kenya [50], isolates from Peru and Colombia mostly invaded via SA-independent receptors (Nr). In contrast, but similar to other studies in India, The Gambia, Tanzania and Senegal [25], [45], [47], [49], [51], most (73%) isolates from Brazil (both Belém and Mato Grosso) were largely dependent on sialylated receptors (Ns). Invasion of most field isolates from South America was independent of chymotrypsin sensitive receptors (Cr). Our findings showing a positive correlation between percentages of invasion into trypsin- and chymotrypsin-treated RBCs are similar to what was shown with Tanzanian isolates [49]. The invasion into neuraminidase-treated RBCs did not correlate with either of the other two enzyme treatments as was reported in studies with Tanzanian [49] and Senegalese isolates [45]. Notably, although the studied areas in South America are hypoendemic for malaria and with low parasite clonality [2], all eight possible invasion profiles were found, which were distributed differentially in the three regions studied. Notably, four major invasion pathways were observed: i) NrTrCr; ii) NsTsCr; iii) NsTrCr; and iv) NrTsCr.

Glycophorin A (GPA) is the dominant sialoglycoprotein on the RBC surface and the EBA-175 receptor [19]. The use of this pathway for invasion is dominant in field isolates from The Gambia [25], but probably not much by isolates from South America based on data presented here. The invasion profile characteristic of EBA-175/GPA was NsTsCr [12], [19], [59], which was found only in one field isolate from Peru and eight isolates from Brazil (Figures 3A and 3B, Table 1), of which only two isolates were unable to invade GPA-lacking, En (a-) RBCs [48]. The role of EBA-140/GPC invasion pathway in the South American isolates, however, needs to be studied since its enzymatic profile is also NsTsCr [20], [21]. In Brazil, Belém and Mato Grosso, NsTrCr was found to be another important invasion pathway. This binding profile was described for PfRh1/receptor Y [30], [40], [48], suggesting that those parasites use, at least in part, this pathway for invasion. The invasion profiles found in field isolates from Colombia and Peru could be broadly characterized as neuraminidase and chymotrypsin resistant, and independent of RBC sensitivity to trypsin treatment, NrTr/sCr. Notably, a similar RBC binding profile was described for PfRh5 [36], [37] and its receptor basigin [39], suggesting that this ligand might be an important part of the invasion machinery employed by field isolates from South America. Preliminary study trying to examine the possible contribution of PfRh5 in RBC invasion by Peruvian parasites using the NrTrCr invasion pathway was performed using a Growth Inhibition Assay (GIA). A Peruvian isolate (F13; NrTrCr) and 3D7 (NsTsCr) laboratory strains were cultured in the presence of 200 µg/ml of anti-PfRh5 (raised against recombinant PfRh5 protein; aa 31–174) or normal purified IgG antibodies. Anti-PfRh5 antibodies significantly inhibited 40% and 46% of invasion by F13 and 3D7, respectively [37]. These findings support the use, at least in part, of PfRh5 for invasion in this Peruvian isolate which is consistent with published studies showing that the PfRh5/basigin invasion pathway is essential for RBC invasion in all tested *P. falciparum* strains [39], [60]. Future GIA studies in all isolates using the NrTrCr pathway will confirm the essential participation of PfRh5 during this novel invasion.

Additional clues for the involvement of PfRh5 and potentially two other ligands in the NrTrCr invasion pathway came from studying the prevalence of specific polymorphisms across all EBL and PfRh family members in the isolates from South America and the association between their prevalence and the use of the NrTrCr pathway. It appeared that some EBL and PfRh variants were more frequently present in isolates from a specific region than others. As noted before, parasites displaying the NrTrCr invasion profile accounts for most of the isolates in Colombia (67%) and Peru (44%). Notably, these particular parasites are also characterized by the prevalent presence of the PfRh2a pepC variant, the PfRh5 variant 1 and EBA-181 RVNKN variant, which are also significantly associated with this invasion profile (Figures S2B, S3C, and S3F). Notably, the EBA-181 polymorphic variants were reported to change receptor specificity, and the EBA-181 RVNKN variant was shown to be associated with NrTrCr RBC binding profile [14]. Moreover, PfRh5 variant 1 was shown to be associated with the NrTr/sCr invasion profile, the two most frequent pathways we found in Colombia and Peru. Unfortunately, there are no studies using the erythrocyte binding assays (EBA) that associated different PfRh variants with specific invasion profiles. Hence future studies using RBC binding assays are needed to confirm whether the PfRh2a pepC variant is also associated with NrTrCr RBC binding profile. Yet, an NrTrCr/s binding profile was described for a 140 kDa-processed product in the C-terminal end of PfRh2a [41]. Furthermore, studying the *ebl* and *Pfrh* expression levels in field isolates displaying the NrTrCr profile by qRT-PCR also pointed to PfRh2a, PfRh5 and EBA-181 as being the major players in this invasion pathway; their corresponding transcripts were the most abundant ligands in the four Peruvian NrTrCr isolates studied (Figure 7A and 7B).

Previously data suggest that the invasion pathway of a particular isolate or strain depends not only on the set of ligands, EBL or PfRh, but also on a hierarchy of molecular interaction that determines which of the expressed ligands is being used [61], [62]. It was reported that the *Pfrh5* gene expression in W2mef**Δ**2a strain was lower in comparison to wild-type W2mef [62] and that PfRh2b functions cooperatively with EBA-181 [9]. Moreover, positive correlations between the expression of PfRh2a/PfRh2b, EBA-175/EBA-140 and PfRh2b/EBA-181 have been also observed in field isolates [49], [51], [63]. We propose that also in parasites from South America that utilize the NrTrCr invasion profile, when each treatment provides only >50% of resistance to invasion, both sets of the EBL and PfRh ligands are utilized for invasion. One set is via the SA-independent ligands, PfRh2a (NrTrCr/s binding profile used by processed form [41]) and PfRh5 (NrTrCr binding profile in 3D7 strain [36], [37]). The other set involves EBA-181, which is known to change its RBC specificity due to amino acid polymorphisms in the binding domain (RVNKN variant binds to NrTrCr RBCs) [14]. The ability of parasites to use EBL and PfRh ligands synergistically supports the hypothesis that a multiplicity of invasion ligands allows *P. falciparum* a more flexible and efficient ability to invade different RBC types in different human populations.

The other invasion profiles that were prevalent in South American isolates were: i) NrTsCr the second most common pathway in Peruvian isolates (25%; Figure 3), which was characterized by the predominant expression of the PfRh2a pepA variant and PfRh5 variant 2; ii) NsTrCr, the second most common invasion pathway in Mato Grosso (36%; Figure 3), that was characterized by predominant expression of the PfRh2a/PfRh2b pepB variant, PfRh5 variant 3, EBA-181 RVIQN variant and the EBA-175 Dd2-like ligand variant, all of which were also significantly associated with the NsTrCr invasion (Figure S2 and S3); iii) NsTsCr, the most common invasion pathway used by parasites from Mato Grosso (50%) (Figure 3) characterized by the PfRh2a pepA variant and PfRh5 variant 2.

As a single enzyme treatment can remove multiple receptors, it is not possible to conclude just based on the sensitivity to treatment the precise usage of any single particular receptor for any invasion pathway. Thus, future studies using the GIA approach with a broader array of antibodies raised against all the EBL and PfRh ligands, the known RBC receptors, and other putative receptors might help to reveal further the molecular basis of the NrTrCr atypical but dominant invasion pathway utilized by the *P. falciparum* field isolates from South America. Analysis of invasion into mutant RBC lacking known receptors or expressing variants of the receptors with NrTrCr profile could be also valuable [64].

Taken together, the present data, along with previously published data, point to the complexity of *P. falciparum* invasion in field isolates. It has long been recognized that the invasion process of RBCs by *P. falciparum* is redundant and a vaccine based on only one invasion ligand is unlikely to overcome the problem of “vaccine resistance” [65]. The present study is directly relevant to asexual blood stage vaccine design focused on invasion pathway proteins, suggesting that regional invasion variants and global geographical variation are likely to preclude a simple one size fits all type of vaccine. The potential use of the EBL and PfRh protein family members in a multi-component vaccine against *P. falciparum* has been proposed [8], [66] and tested *in vitro* [9], [67]. Our study and other studies also highlight the importance of studying polymorphisms in the EBL and PfRh family members as well as their expression levels in all endemic regions to further confirm if the associations between ligand polymorphisms and invasion profiles are region-specific. Of particular interest will be to include in future studies also polymorphisms in the binding domains of the PfRh ligand members and analyze whether variants in the binding domains are in linkage disequilibrium with the other regions, which could explain why we find association between invasion and polymorphisms in the non-binding domains. Preliminary studies with the South American isolates indicated that some of these polymorphisms (in particular those in PfRh1 and PfRh2) cluster regionally and are in part novel. The finding that the NrTrCr invasion pathway is more common in Colombian and Peruvian field isolates than in other regions argues that further research to identify the molecular interactions underlying this pathway is of major importance, in particular if the malaria vaccines are aimed worldwide.

## Materials and Methods

### Ethics Statement

The research was approved by the Ethics Committee of the Centro de Investigaciones Medicas, Facultad de Medicina, Universidad de Antioquia, Colombia; Universidad Peruana Cayetano Heredia Institutional Review Board (IRB), Peru; Instituto Evandro Chagas IRB, Brazil; and by the New York Blood Center’s IRB.

### Study Sites

*P. falciparum* parasites were collected in three South American countries (Figure 1): i) Urabá, Colombia, a region of the Antioquia state located on Urabá Gulf in the Caribbean Sea, near the borders with Panamá. Most of the inhabitants are mestizos but there is well-known African admixture in this region, but also there are some indigenous communities that appear to be without such admixture. The whole population is exposed to malaria transmission, which is unstable with a hypoendemic pattern [2], [68]; ii) Loreto, Peru, located in the Northern part of the Peruvian Jungle (Amazon Region). It covers 30% of the national territory, and comprises parts of the High and Low Jungle. The inhabitants of this region are Hispanic/Indian mestizos and indigenous people without evidence of African admixture. Malaria transmission in this area is hypoendemic and unstable with a peak during the rainy season [2]; and iii) Belém, North of Brazil, located on the Amazon River with a tropical forest. Most of the inhabitants are interethnic mix between Native-American, Black and Caucasian populations. The area is characterized by a hypoendemic pattern of malaria transmission, due mainly to its low demographic index [69]. The samples from Mato Grosso, Brazil were previously reported and characterized [48].

### *P. falciparum* Field Isolates

Parasites used in this study were collected from consenting patients with uncomplicated malaria from Colombia (n = 6; Urabá region, 2006), Peru (n = 16; Loreto, 2008–2010) and Brazil (n = 8; Belém, 1992–2009). In addition, we had access to three adapted fresh isolates from Peru (PC15, PC26 and PC49; kindly provided by Dr. Xinzhuan Su, NIH) that were collected in 1997–1998 in Padre Cocha, Loreto State in Peru. After venous blood samples were collected the buffy coat was removed and the parasitized red blood cells (pRBC) were cryopreserved in liquid nitrogen using glycerolyte solution. The field isolates from Belém, Brazil were assayed for invasion in Belém (24.2% success rate for long-term cultures), and the field isolates from Peru and Colombia were assayed for invasion in New York (93.75% and 85.7% success rate for long-term cultures, respectively). All assays were done by using standard reagents and protocols and by personnel from the New York Blood Center using identical techniques with identical training. Field isolates were assay thawed and cultured in complete RPMI medium (RPMI 1640 medium containing 2 mM L-glutamine, 25 mM HEPES, 3.42 mM hypoxanthine, 2 g/L Glucose, 3.26 µM GSH, 25 µg of gentamicin/ml and 21.6 mM NaHCO_3_) supplemented with 10% inactivated pooled human A positive serum. Parasites were cultured for approximately two weeks before they were used in the invasion assays. Parasites cultures were maintained in A positive RBCs at a 5% hematocrit at 37°C with a gas mixture of 5% CO_2_, 5% O_2_, and 90% N_2_. Five-laboratory strains were grown in a continuous culture by standard techniques [70]. *P. falciparum* HB3 is a clone from Honduras I/CDC strain (HB3; MRA-155, MR4, ATCC Manassas Virginia) [71]; 7G8 is a clone of the Brazilian strain IMTM22 (7G8; MRA-926, MR4, ATCC Manassas Virginia) [72]; 3D7 is a clone from NF54 isolate, imported to the Netherlands from West Africa [73]; W2mef was selected from clone W2 (Indochina) for resistance to mefloquine [74]; and Dd2 is a clone derived from W2mef [75], which appeared in our study and others to utilize SA-dependent RBC invasion. Malaria Group, Universidad de Antioquia, Colombia, provided the DNA from the Colombian FCB-2 laboratory strain.

### Enzymatic Treatment of RBCs

Whole blood was obtained from consenting healthy volunteer donors at New York Blood Center, New York; Belém, Brazil and Lima, Peru. Fresh blood (A positive, MNS positive as determined by agglutination with monoclonal antibodies; see below) was washed three times in incomplete RPMI medium and twice in phosphate-buffered saline (PBS) to remove the buffy coat and plasma before treatment with enzymes. Briefly, 0.1 ml of packed RBCs (approximately 10^9^ cells) were treated with either 0.1 ml of 0.1 IU/ml neuraminidase (Sigma) in PBS pH 7.3; 1 ml of 10 mg/mL of TPCK-treated trypsin (N-*p*-Tosyl-L-phenylalanine chloromethylketone; Sigma) in PBS pH 7.7, or 1 ml of 2 mg/ml TLCK-treated chymotrypsin (N-α-*p*-Tosyl-L-lysine chloromethylketone; Sigma), in PBS pH 8.0. After 30 min at 37°C, with periodic shaking, the cells were washed three times with PBS. Then, 1 mg/ml soybean trypsin inhibitor (Sigma) was added to the trypsin-treated RBCs and the mixture was shaken for 10 min at room temperature to inactivate the enzyme. The treated RBCs were washed three times with PBS and resuspended at a 50% hematocrit in incomplete RPMI medium before use in the invasion assays. Untreated RBCs (control) were incubated and washed in the same way as the enzyme-treated cells. The enzyme-treated and untreated RBCs were stored at 4°C and used within 24 h after treatment.

### Efficacy of Enzymatic Treatments

For assessment of the efficacy of neuraminidase treatment, peanut lectin (*Arachishypogaea*; E Y laboratories) was used to detect the T antigen, which is exposed after SA residues had been cleaved by neuraminidase treatment of the RBCs. For assessment of the efficacy of trypsin and chymotrypsin treatment, anti-M and anti-S antibodies were used, respectively (provided by Gregory Halverson Laboratory of Immunochemistry, New York Blood Center, New York). The M antigen is present on GPA, which is cleaved by trypsin at residues 31 and 39, leading to loss of the M antigen. Similarly, the S antigen is present on GPB and is cleaved by chymotrypsin at residue 34, leading to loss of this antigen. A 10 µl aliquot of the enzyme-treated RBCs was suspended at a 5% hematocrit in PBS pH 7.4. Fifteen microliters of each treated RBCs were added to the same volume of peanut lectin or 30 µl of anti-M or anti-S antibodies in a glass tube. The samples were mixed and incubated for 5 min at room temperature. The samples were then centrifuged before the agglutination was assessed visually and scored on a scale of (–) to (+++), where (–) is no agglutination and (+++) is maximum agglutination. All three enzymes worked efficiently since treatment with neuraminidase, trypsin and chymotrypsin resulted in the loss of SA residues as well as the M and S antigens, respectively.

### RBC Invasion Assays

*P. falciparum* trophozoites were obtained after two rounds of synchronization using 5% sorbitol treatment [76] followed by purification using the Percoll/sorbitol gradient method [77]. Parasitized RBCs with mature stages (>95% purity) were washed twice in incomplete RPMI medium and added to untreated or enzyme-treated RBCs in duplicate wells of a 12-well plates to yield a 0.5–1% parasitemia and 2% hematocrit in a total volume of 500 µl of complete RPMI medium. The plates were incubated at 37°C for 20 h using a gas mixture of 5% CO_2_, 5% O_2_, and 90% N_2_ within a modular incubator chamber. The pre-invasion and the ring-parasitized RBCs after 20 h incubation were analyzed for parasitemia using thin blood smear stained with Giemsa. The percentage of pRBC was determined by counting at least 1,000 RBCs in each of the two slides taken from each well. Parasite multiplication rate (PMR) was determined by dividing the number of ring-parasitized RBC after invasion by the pre-invasion parasitemia. Successful invasion was defined as two-fold initial parasitemia (greater than 1–2% post-invasion) into untreated RBC. Invasion efficiencies into enzyme-treated RBCs were expressed as percentages of invasion relative to invasion into untreated RBCs. Parasites were grouped into invasion profiles based on their sensitivity to enzyme treatment: neuraminidase (N), trypsin (T) and chymotrypsin (C). If the percentage of invasion was less than 50% of the untreated RBCs, the isolate was considered to be sensitive to that particular treatment. If the percentage of invasion was more than 50% of the untreated RBCs, the isolate was considered to be resistant to that particular treatment. Thus, the invasion profiles were defined as all eight options of Nr/sTr/sCr/s. The invasion of all field isolates as well as the laboratory strain controls was assessed in two to four independent experiments.

Although the original published invasion study of the Mato Grosso isolates used a seven day assay in the presence of target RBCs [48], unpublished repeated invasion assays with these adapted parasites using the 20 h invasion protocol verified that the invasion profiles are the same, and thus could be used for our comparative analyses.

### Genotyping of *P. falciparum* Field Isolates

Genomic DNA (gDNA) was extracted and purified from 200 µl of packed pRBC using QIAamp DNA Blood Mini Kit according to manufacturer’s instructions (Qiagen, CA, USA), eluted using buffer AE and stored at −20°C for PCR analysis. The presence of single or multiple genotypes in the field isolates was determined by PCR amplification of two polymorphic loci, *msp-1* block 2 and *msp-2* block 3. Oligonucleotide primers based on conserved sequences flanking these polymorphic regions were used as previously reported [48]. When possible, the PCR typing was performed on the frozen samples collected from patients and after the parasites had been cultured, with no evident changes in the genotypes indicating that the assays were performed on the same isolates collected from the patients. Only one sample from Colombia had two detectable genotypes and was not included in this study.

### Sequencing of the Polymorphic Regions of the EBL and the PfRh Ligand Proteins

Genomic DNA from field isolates collected in Colombia, Peru and Belém, Brazil was used as template for amplification and sequencing of the polymorphic region of *ebl* and *Pfrh* genes using specific primers designed based on reference sequences of laboratory strains (Table S1). Sequences of six laboratory strains from different geographical origins (3D7, 7G8, HB3, Dd2, W2mef and FCB-2) were also obtained. DNA from the field isolates from Mato Grosso, Brazil [48] were also used to sequence regions of *eba-175*, *ebl-1*, *Pfrh5* and to determine if the *Pfrh2b* large deletion was present. PCR amplification was performed with 100 ng of template DNA using Platinum® PCR SuperMix High Fidelity (Invitrogen). PCR products were purified using a QuickClean II PCR Purification Kit (GenScript, Inc) and sent for custom direct sequencing procedures (GENEWIZ, Inc) using specific primers (Table S1). The ClustalW2 alignments (http://www.ebi.ac.uk/Tools/msa/clustalw2/) of the corresponding amino acid sequences against the reference sequences were utilized to determine polymorphisms.

### RNA Extraction and Reverse Transcription

Samples for *P. falciparum* RNA preparation were obtained of highly synchronized cultures at the schizont stage. The pRBC pellet was treated with 0.2% saponine at room temperature, washed once in PBS and the RNA was extracted using High Pure RNA Isolation Kit (Roche) according to manufacturer’s instructions. Aliquots were stored at −80°C for subsequent complementary DNA (cDNA) transcription. A total of 2 µg of RNA was reverse-transcribed with random hexamer primers using Transcriptor first strand cDNA Synthesis Kit (Roche).

### Quantitative Real TimePCR (qRT-PCR) Analysis

Specific primers (Table S2) for the *Pfrh1*, *Pfrh2a*, *Pfrh2b*, *Pfrh4* [63], *Pfrh5* [51] and *eba-175* [78] genes were prepared and used for qRT-PCR analysis as described. Primers for *eba-181* and *18S rRNA* were designed in our laboratory using Primer Express software (Applied Biosystems, Carlsbad, CA, USA). For relative quantification of the different genes, for each transcript triplicate qRT-PCR reactions were performed using LightCycler® 480 DNA SYBR Green I Master (Roche) on a LightCycler480® II System (Roche). Threshold cycle (Ct) values were obtained and the relative changes in gene expression of each transcript in the “test” *versus* “control” isolate were calculated with the 2^−ΔΔCt^ method using endogenous *18S rRNA* housekeeping gene [79]. The fold change of each of the *ebl* and *Pfrh* genes was then normalized as a proportion of the sum of the fold change for the seven genes in each isolate, and the data presented as the relative proportion for each gene.

### Statistical Analysis

The Mann-Whitney U or the Kruskal-Wall tests were used to assess the differences between two or more groups, respectively. Dunn’s Mutiple Comparison Test was used as post-hoc analysis. Spearman’s rank correlation (r_s_) was used to assess the correlation between invasion efficiencies into enzyme-treated RBCs. Fisher’s exact test was used to compare the proportion of isolates using a specific invasion pathway in the different countries. A *p* value <0.05 was considered significant. Statistical analysis was performed using GraphPad Prism version 4.0 software (GraphPad Software, Inc. CA, USA).

Principal Component Analysis (PCA) was used to gain additional insights on the potential association between specific invasion pathways and the EBL/PfRh polymorphisms. PCA is a multivariate statistical method which allows the representation of the original dataset in a new reference system characterized by new variables, called principal components (PCs), which are obtained by the linear combination of the original ones. The original purpose of PCA is to reduce a large number (*p*) of variables to a much smaller number (*m*) of PCs whilst retaining as much as possible of the variation in the *p* original variables. Its power as an analytical tool is to generate new hypotheses, which could be then verified experimentally [80]. In this study, two PCs were obtained from analyzing the three variables that describe the invasion profiles; neuraminidase, trypsin and chymotrypsin treatment. The two components generated explained 82% of the total variance and were used to plot the distribution of the isolates based on their sensitivities to the enzyme treatments. The first PC includes the variables trypsin and chymotrypsin treatment. The second PC includes the variable neuraminidase treatment. In the PCA projection, parasites that are more sensitive to the enzymatic treatment are within the negative coordinates and those that are more resistant to the treatment cluster within the positive coordinates. PCA were projected using a total of 50 samples, including field isolates from Colombia, Peru and Belém, those from Mato Grosso, reported in our previous study [48], and five laboratory strains. To reveal potential association between defined polymorphisms and the specific invasion pathways, contingency tables (2×2) were created and Fisher’s exact test was used to calculate a *p* value. A *p* value<0.05 was considered significant. The PCA was performed using SPSS version 10.0 software (SPSS Inc., Chicago, IL, USA).

## Supporting Information

Figure S1 **Correlation of invasion efficiency into enzyme-treated erythrocytes by field isolates from Colombia, Peru and Belem, Brazil.** (**A**) Percentage of invasion into neuraminidase-treated RBCs compared to trypsin-treated RBCs. (**B**) Percentage of invasion into neuraminidase-treated RBCs compared to chymotrypsin-treated RBCs. (**C**) Percentage of invasion into trypsin-treated RBCs compared to chymotrypsin-treated RBCs. Spearman correlation coefficient, abbreviated r_s_, and *p* value are shown.
(TIF)

Figure S2 **Association between polymorphisms in EBL ligands and invasion profiles.** Principal component analysis was obtained using data from invasion assays and their sensitivities to treatment with neuraminidase (N), trypsin (T) or chymotrypsin (C). The first and second principal component coordinates reflect the trypsin/chymotrypsin and neuraminidase sensitivities, respectively. In both PC coordinates, isolates that are more sensitive to the treatment cluster within the negative coordinates and isolates that are more resistant cluster within the positive coordinates. (**A**) Invasion profiles displayed by the field isolates from South America. (**B**) Association analysis for EBA-181. Note the association between RVIQN variant and the NsTrCr invasion profile and between the RVNKN variant and the NrTrCr invasion profile. (**C**) Association analysis for EBA-175. Note the association between variant 4 and the NsTrCr invasion profile. (**D**) Association analysis for EBL-1. Note the association between the *ebl-1* gene sequence containing the 5 T’s insertion and the NrTrCr invasion profile. The polymorphisms in the EBA-181, EBA-175 and EBL-1 are based on those presented in the Figure 4.
(TIF)

Figure S3 **Association between polymorphisms in PfRhligands and invasion profile.** Principal component analysis was obtained using data from invasion assays and their sensitivities to treatment with neuraminidase (N), trypsin (T) or chymotrypsin (C). The first and second principal component coordinates reflect the trypsin/chymotrypsin and neuraminidase sensitivities, respectively. (**A**) Invasion profiles displayed by the field isolates from South America. (**B**) Association analysis for PfRh1.Note the association between parasites containing the 10 aa deletion (10D and 10D* codes) and the TrCr profile. (**C**) Association analysis for PfRh2a. Note the association between the pepB variant (B in the graph) with the NsTrCr invasion profile and pepC with the NrTrCr invasion profile. (**D**) Association analysis for PfRh2b. Note the association between the NsTrCr invasion pathway and pepB variant, while pepC* was associated with the Nr/sTsCr/s invasion pathway. (**E**) Association analysis for PfRh4. Note the association DEVE modified (codes 1+1 or 1+2) and NsTsCs profile. (**F**) Association analysis for PfRh5. Note the association between the NsTrCr invasion profile and variant 3, whereas variant 1 is associated with the NrTs/rCr invasion profile. The polymorphisms in PfRh1 (**B**), PfRh2a (**C**), PfRh2b (**D**), PfRh4 (**E**) and PfRh5 (**F**) are based on those presented in the Figure 5 and Figure 6.
(TIF)

Table S1 **Sequences of primers used for PCR and sequencing.**
(DOC)

Table S2 **Sequences of primers used for quantitative real time PCR (qRT-PCR).**
(DOC)
