# Supplementary figures and images for: Plasmodium falciparum Field Isolates from South America Use an Atypical Red Blood Cell Invasion Pathway Associated with Invasion Ligand Polymorphisms
Source: PLoS One. 2012 Oct 31;7(10):e47913. doi: 10.1371/journal.pone.0047913 (PMC3485327; doi:10.1371/journal.pone.0047913)

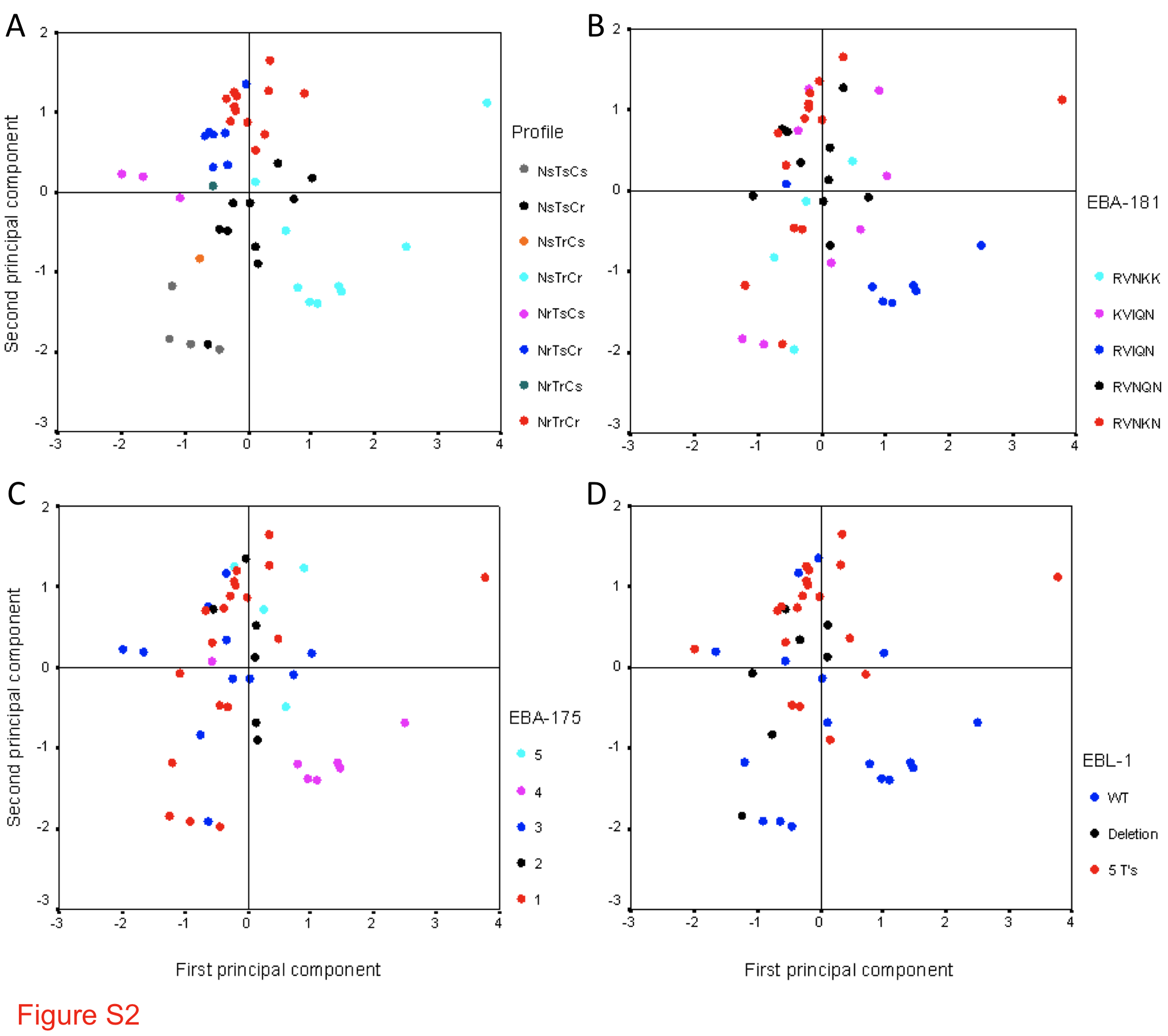

Supplement: Figure S2 — Association between polymorphisms in EBL ligands and invasion profiles. Principal component analysis was obtained using data from invasion assays and their sensitivities to treatment with neuraminidase (N), trypsin (T) or chymotrypsin (C). The first and second principal component coordinates reflect the trypsin/chymotrypsin and neuraminidase sensitivities, respectively. In both PC coordinates, isolates that are more sensitive to the treatment cluster within the negative coordinates and isolates that are more resistant cluster within the positive coordinates. (A) Invasion profiles displayed by the field isolates from South America. (B) Association analysis for EBA-181. Note the association between RVIQN variant and the NsTrCr invasion profile and between the RVNKN variant and the NrTrCr invasion profile. (C) Association analysis for EBA-175. Note the association between variant 4 and the NsTrCr invasion profile. (D) Association analysis for EBL-1. Note the association between the ebl-1 gene sequence containing the 5 T’s insertion and the NrTrCr invasion profile. The polymorphisms in the EBA-181, EBA-175 and EBL-1 are based on those presented in the Figure 4. (TIF) [file pone.0047913.s002.tif]

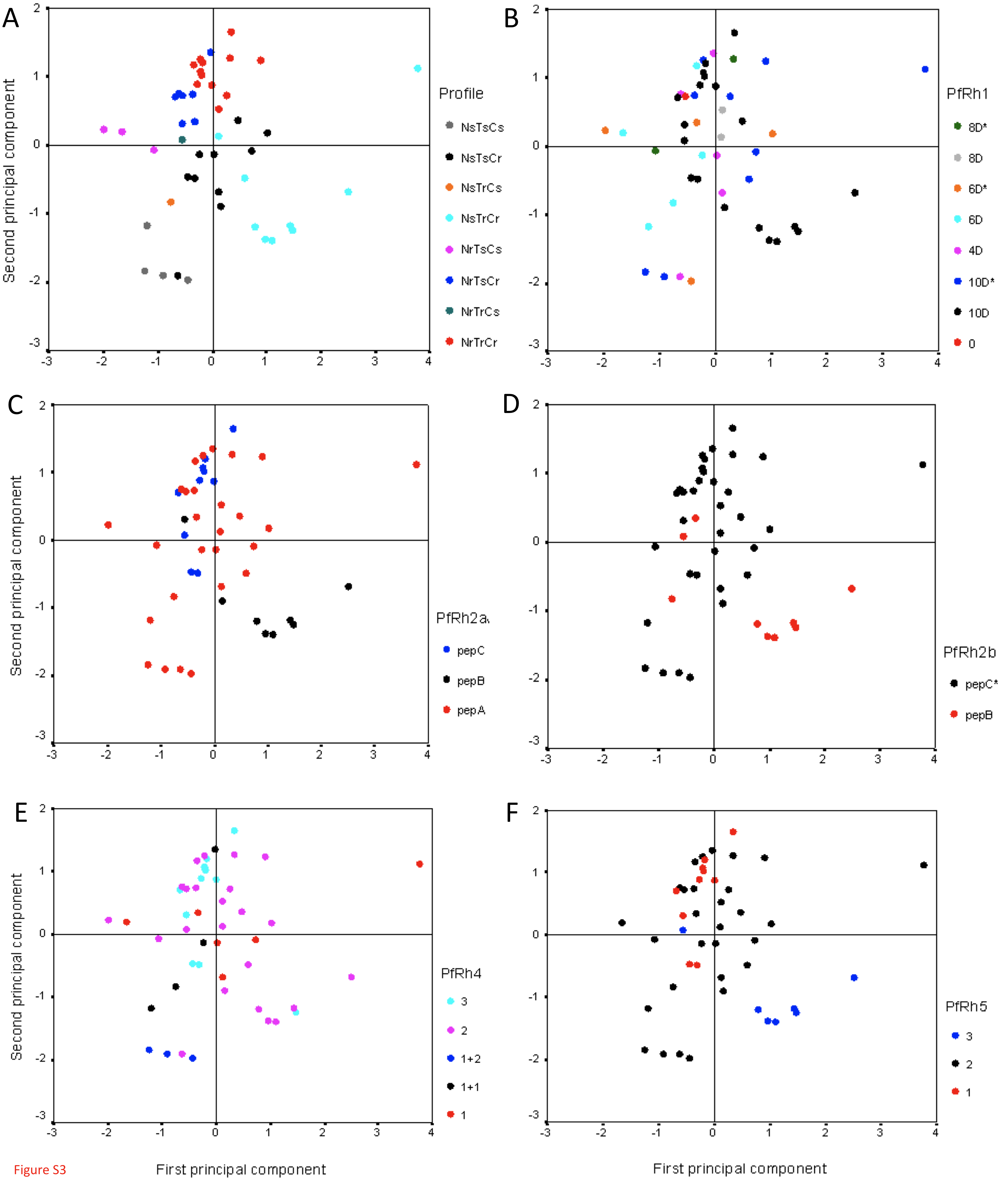

Supplement: Figure S3 — Association between polymorphisms in PfRhligands and invasion profile. Principal component analysis was obtained using data from invasion assays and their sensitivities to treatment with neuraminidase (N), trypsin (T) or chymotrypsin (C). The first and second principal component coordinates reflect the trypsin/chymotrypsin and neuraminidase sensitivities, respectively. (A) Invasion profiles displayed by the field isolates from South America. (B) Association analysis for PfRh1.Note the association between parasites containing the 10 aa deletion (10D and 10D* codes) and the TrCr profile. (C) Association analysis for PfRh2a. Note the association between the pepB variant (B in the graph) with the NsTrCr invasion profile and pepC with the NrTrCr invasion profile. (D) Association analysis for PfRh2b. Note the association between the NsTrCr invasion pathway and pepB variant, while pepC* was associated with the Nr/sTsCr/s invasion pathway. (E) Association analysis for PfRh4. Note the association DEVE modified (codes 1+1 or 1+2) and NsTsCs profile. (F) Association analysis for PfRh5. Note the association between the NsTrCr invasion profile and variant 3, whereas variant 1 is associated with the NrTs/rCr invasion profile. The polymorphisms in PfRh1 (B), PfRh2a (C), PfRh2b (D), PfRh4 (E) and PfRh5 (F) are based on those presented in the Figure 5 and Figure 6. (TIF) [file pone.0047913.s003.tif]
