# Supplementary material for: Plasmodium falciparum Field Isolates from South America Use an Atypical Red Blood Cell Invasion Pathway Associated with Invasion Ligand Polymorphisms
Source: PLoS One. 2012 Oct 31;7(10):e47913. doi: 10.1371/journal.pone.0047913 (PMC3485327; doi:10.1371/journal.pone.0047913)
Supplement: Table S1 — Sequences of primers used for PCR and sequencing. (DOC) [file pone.0047913.s004.doc]

**Table S1.** Sequences of primers used for PCR and sequencing.

| **Primers used for PCR amplification of EBL and PfRh genes** | | | | |
| --- | --- | --- | --- | --- |
| **Gene** | **GenBank** | **Primer** | **Sequence (5'- > 3')** | **Ref** |
| **name** | **accession no.** | **name** |  |  |
| *Pfrh1* | AF411930 | Rh1-OF | ATCTAATTCATGTTAAGAAACAATTTGAACACACC | [44] |
|  |  | Rh1-OR | TAGATATATCTTGTTCCTGTAATTTTGTTG |  |
| *Pfrh2a* | AY138497 | Rh2a-OF | AACACTTGAATCAATTCAAACG | [44] |
|  |  | Rh2a-OR | ATGATTTCATCCTTCTCC |  |
| *Pfrh2b* | AY138500 | Rh2b-OF | AACACTTGAATCAATTCAAACG | [44] |
|  |  | Rh2b-OR | TGTGTTTCCATAGGTTCATCAAGTG |  |
| *Pfrh4* | AF432854 | Rh4-2F | TAGTGAGACAAATGACATGAT | [44] |
|  |  | Rh4-2R | TCATATGTCATTAAAATCTTC |  |
| *Pfrh5* | PFD1145c | Rh5-OF | CAGGATTAAGTTTTGAAAATGC | [35] |
|  |  | Rh5-OR | CCATGTTTTGTCATTTCATTG |  |
| *eba-175* | FJ655429 | EBA175 F2-F | GTTGATACAAACACAAAGGTG | [25] |
|  |  | EBA175 F2-R | CCTTTACTTCTGGACACATCG |  |
| *eba-181* | AF461096 | EBA181-OF | GGTAGGAAAGGGGAATATTTGAAT | [48] |
|  |  | EBA181-OR | ACCACTTGCGACACTCACAGAATTGTG |  |
| *ebl-1* | AF131999 | EBL1-OF | atgaatgtacccCTGAATATAAAGTTCCTT |  |
|  |  | EBL1-OR | GTATTCGTCTTATTGGGGC |  |
| **Primers used for sequencing of EBL and PfRh genes** | | | | |
| **Gene** | **GenBank** | **Primer** | **Sequence (5'- > 3')** | **Ref** |
| **name** | **accession no.** | **name** |  |  |
| *Pfrh1* | AF411930 | Rh1-1Fseq | CAACATGATAATCAAAATGTTG |  |
| *Pfrh2a* | AY138497 | Rh2a-3F | GAAAGAGAAAGATTGGAAAAAGCG | [44] |
|  |  | Rh2a-5F | TTATTTCAAATGGAAGAGATG |  |
| *Pfrh2b* | AY138500 | Rh2a-3F | GAAAGAGAAAGATTGGAAAAAGCG | [44] |
|  |  | Rh2a-5F | TTATTTCAAATGGAAGAGATG |  |
| *ebl-1* | AF131999 | EBL1-2F | CAGACAGTGATGAAAGATTTTCT |  |
