# Supplementary material for: Plasmodium falciparum Field Isolates from South America Use an Atypical Red Blood Cell Invasion Pathway Associated with Invasion Ligand Polymorphisms
Source: PLoS One. 2012 Oct 31;7(10):e47913. doi: 10.1371/journal.pone.0047913 (PMC3485327; doi:10.1371/journal.pone.0047913)
Supplement: Table S2 — Sequences of primers used for quantitative real time PCR (qRT-PCR). (DOC) [file pone.0047913.s005.doc]

**Table S2. Sequences of primers used for quantitative real time PCR (qRT-PCR)**

| **Primers used in qRT-PCR of EBL and PfRh genes** | | | | |
| --- | --- | --- | --- | --- |
| **Gene** | **GenBank** | **Primer** | **Sequence (5'- > 3')** | **Ref** |
| **name** | **accession no.** | **name** |  |  |
| *Pfrh1* | AF533700 | Rh1-F RT | GCAAAGGTGGATTTTCTGCAACAT | [63] |
|  |  | Rh1-R RT | TCTGAATAGTCCTTTCTTATTTTTTCATTGGAAGA |  |
| *Pfrh2a* | AY138496 | Rh2a-F RT | GGTCATAAAAGATAATGAGACACAATTGAAAACAA | [63] |
|  |  | Rh2a-R RT | GCAATATTTTTTTCTGGTTTTCTTCGTATTCC |  |
| *Pfrh2b* | AY138500 | Rh2b-F RT | ACAGAAAGCGATGATATTGATAACAGTGAA | [63] |
|  |  | Rh2b-R RT | CCCATGGGTGTTACTTCTATGACT |  |
| *Pfrh4* | AF432854 | Rh4-F RT | GAAATGACGCAATTCCCTCAAAAGA |  |
|  |  | Rh4-R RT | GGTGTGTTTTATTTATATCATGTTGATTCTGTGA | [63] |
| *Pfrh5* | PFD1145c | Rh5-F RT | ACGAAGAATCAAGAAAATAATCTGACGTTACT |  |
|  |  | Rh5-R RT | TGTTGAATGATCTTTAGCATTATTTGTTTTTATATTCTCTTT | [51] |
| *eba-175* | FJ655429 | eba175-F RT | AATTTCTGTAAAATATTGTGACCATATG | [78] |
|  |  | eba175-R RT | GATACTGCACAACACAGATTTCTTG |  |
| *eba-181* | AY496955 | EBA181-Fq | GCGGGTAGTACAATATTAGATGATTC |  |
|  |  | EBA181-Rq | TGTTGTGTGCTAAAATTATGTTCTTG |  |
| *18S rRNA* | 9221844 | 18SrRNA-F | GCTGACTACGTCCCTGCCC |  |
|  |  | 18SrRNA-R | ACAATTCATCATATCTTTCAATCGGTA |  |
